# Venus nightside surface temperature

**DOI:** 10.1038/s41598-018-38117-x

**Published:** 2019-02-04

**Authors:** D. Singh

**Affiliations:** Physical Research Laboratory, Ahmedabad, India

## Abstract

First global map of Venus nightside surface temperature using Akatsuki infrared measurements reveals hot Venus surface with an average surface temperature of about 698 K. Surface temperatures do not show any significant variation with changing latitudes because only a small amount (~2.5%) of solar energy reaches the surface. Surface temperatures are relatively colder at higher altitude regions as compared to lower altitude regions. However, the major temperature variation on Venus surface is governed by various lithospheric heat transport mechanisms. On a global scale, surface temperatures show a spatial variation of about 230 K.

## Introduction

In our solar system, Venus is the second planet and orbiting at a distance of about 0.72 AU from Sun. Venus orbital period (a year) lasts about 225 Earth days, and a sidereal day (rotation in retrograde motion) lasts about 243 Earth days^1^. Venus is similar to Earth in terms of size (0.95 of Earth’s radius), mass (0.814 of Earth’s), bulk density (0.95 of Earth’s), and gravity (0.907 of Earth’s). Due to these similarities Venus is considered as ‘twin-planet’ of Earth. However, Venus also possesses many dissimilarities with Earth such as lack of any intrinsic magnetic field^2,3^, high surface pressure (~93 bar), atmospheric composition (predominantly CO_2_), and high surface temperatures^1^. I focus this work in retrieving Venus surface temperature to improve our understanding, and to constrain its variability.

Measurement of the surface temperature of Venus is one of the primary objectives of Akatsuki mission (also called “Venus Climate Orbiter”)^4^. The 1 μm camera also known as IR1^5^ detects thermal emission at 0.90, 0.97 and 1.01 μm channels. I focus my work on retrieving nightside surface temperatures of Venus because 0.90 μm dayside channel detects solar radiation due to cloud scattering^5,6^. I utilize observations only from 1.01 μm channel because it has highest signal-to-noise ratio^6^ among all three nighttime channels. Also, more than 95% of the radiation measured in IR1 window comes from Venus surface^7^, and the interference caused by cloud inhomogeneity appears to be insignificant^6^.

## Nightside Surface Temperature Of Venus

**Figure 1:**
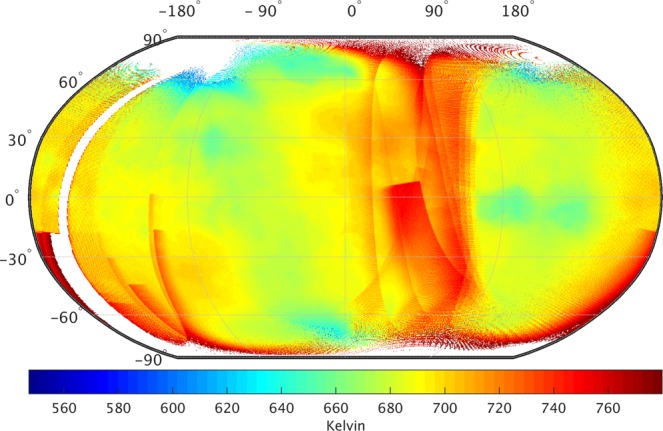
Nightside surface temperature of Venus with spatial resolution of 0.5° × 0.5°. Surface temperature retrievals are based on IR1 data from Akatsuki mission between July 21, 2016 and December 7, 2016. White areas indicate either data is absent or erroneous. High altitude regions are relatively colder than low altitude regions (Data source: https://darts.jaxa.jp/planet/project/akatsuki/ir1.html.en)^6,25^.

Figure 1 shows a global map of nightside surface temperature of Venus at 0.5° × 0.5° spatial resolution. I utilize Level l2b data (https://darts.jaxa.jp/planet/project/akatsuki/) collected after July 21, 2016 (see more details in methods) to generate Fig. 1 gridded map. The global average nightside surface temperature of Venus is about 698 K with a spatial variation of over 230 K. Due to very thick and reflective atmosphere^7–10^, Venus surface absorbs about 2.5% of incident solar flux as compared to about 50% on Earth^11^. Therefore, the surface temperatures do no show a significant variation with latitudes. Relatively high temperatures in 0–90° longitudinal band are probably caused either due to large instrumental bias^6^ or presence of significantly large hot-spots/volcanoes^12–16^. The exact cause for such high temperatures could not be assessed due to very limited availability of useful data.

**Figure 2:**
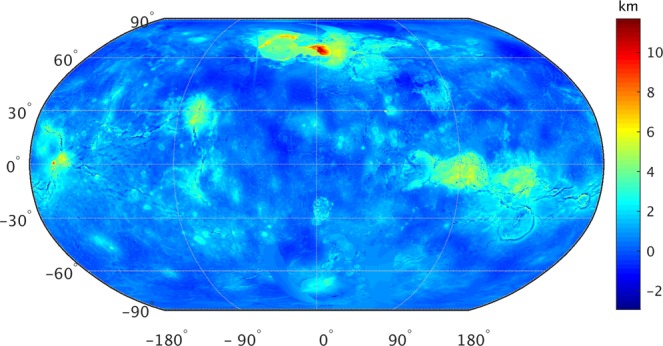
Global map of surface topography (spatial resolution of 0.5° × 0.5°) of Venus based on Magellan radar altimeter data. Vast majority of the planet is covered with flat plains. Most prominent highlands such as Ishtar terra (60–70°N, −60–30°E), Aphrodite terra (along the equator, 60–120°E), Alta Regio (along the equator, −170–−150°N), and Beta Regio (around 30°N, −90°E) are distinctly visible on the map. (Data source: http://pds-geosciences.wustl.edu/missions/magellan/gxdr/).

In general, the retrieved temperatures show a direct dependency on altitude. On average, highlands (altitude >2 km) are colder by about 3 K as compared to lowlands (altitude <0 km). I use Magellan altimeter data^17,18^ to create Venus surface topography map (Fig. 2). Two regions which distinctly show elevation impact on temperature variations are first, Ishtar Terra (located in the Northern Hemisphere) and second, Aphrodite Terra (located along the equator). The highest point on Venus (~11 km), the mountain Maxwell Montes is located on Ishtar Terra. Regions with altitude range from 0 to 2 km have an average surface temperature of about 694 K (similar to global average), substantiating the fact that about 70–80% of Venus surface is covered by regional flat plains^18–20^.

The temperature lapse rate in planetary atmosphere is given by:

1 $$\frac{dT}{dz}=-\frac{g}{{C}_{p}}$$

where, *‘g*’ is acceleration due to gravity, and *‘C*_*p*_*’* is heat capacity at constant pressure. Using Venus physical parameters (*g* = 8.87 m s^−2^ & *C*_*p*_ = 1.16 kJ Kg^−1^ K^−1^), we get a lapse rate (dT/dz) of about 7.6 K km^−1^.

**Figure 3:**
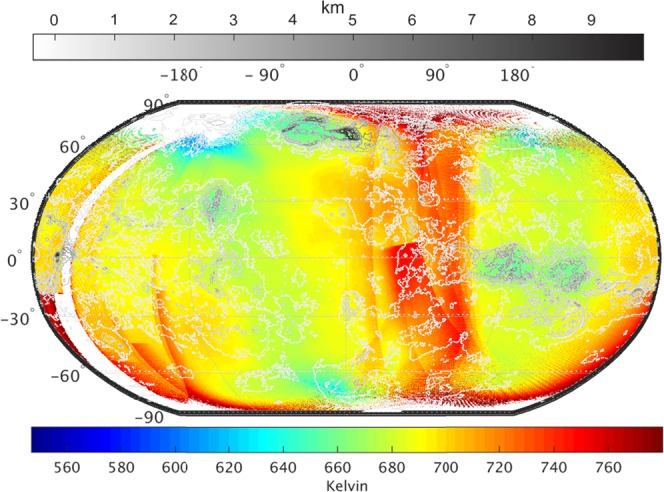
Surface temperature (Fig. 1) merged with topography (Fig. 2). Color scale at the bottom (Kelvin) corresponds to surface temperature variation and grayscale at the top (km) corresponds to topography variation. All the higher altitude regions Ishtar terra, Aphrodite terra, Alta Regio, and Beta Regio are distinctly stand out for relatively lower temperatures as compared to lower altitude regions.

Lapse rates are used to estimate a general trend of temperature variation in a planetary atmosphere. For about 13 km change in Venus topography, a temperature change of about 100 K is expected due to lapse rate only. Merged map of surface temperature and topography (Fig. 3) visually enhances the correlation between altitude and temperature variation. The higher altitude regions are relatively colder as compared to lower altitude regions (Fig. 3). Surface temperature has a tendency to be cool with altitude due to adiabatic lapse rate governing surrounding air temperature. However, this trend is not consistent on Venus as Venusian surface temperatures are mostly controlled by various internal planetary mechanisms rather than by surrounding air.

**Figure 4:**
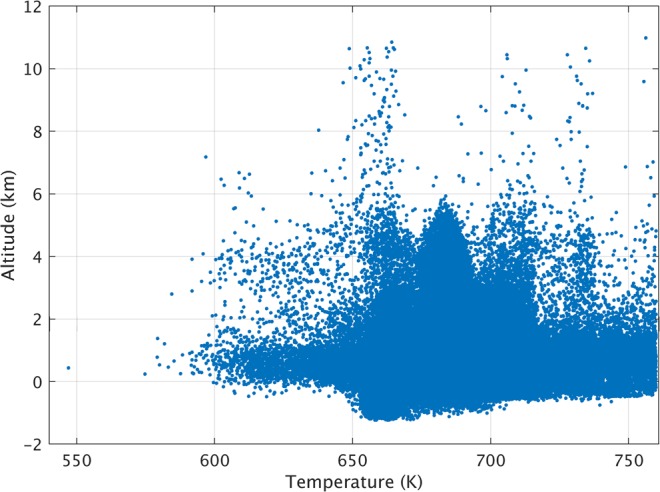
Scatter plot of surface temperature versus altitude. Relatively high temperatures in 0–90° longitudinal band and near poles are excluded from this plot.

The lithospheric heat transport on Venus can occur mainly due to (1) hot-spot volcanism, (2) plate recycling, and (3) lithospheric conduction^21–23^. Depending on the extent/efficiency of each process, different regions of Venusian surface indicate different temperatures. Since most of Venus surface is covered by regional flat plains, the entire surface temperature range occurs within 2 km of zero altitude surface (Fig. 4). Figure 4 also indicates that no strong correlation can be observed between temperature change and altitude. Therefore, the variation of temperature is not only altitude dependent, but can also change due to many other factors such as solar insolation, volcanic activity, greenhouse effect, lithospheric movements, and wind movements.

**Figure 5:**
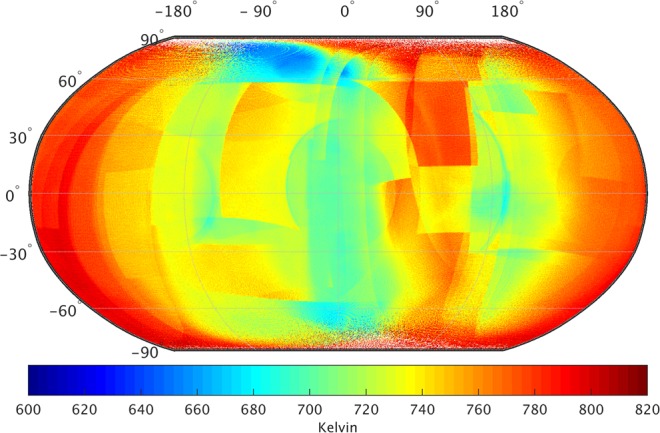
Surface temperature retrievals of Venus based on IR1 instrument of Akatsuki mission between January 1, 2016 and July 21, 2016. Temperatures are mostly washed out and relatively higher than Fig. 1 due to contamination from dayside.

Surface temperature retrievals before July 21, 2016 are not of much use for any analysis as they are mostly washed out due to contamination from dayside (Fig. 5). However, high temperature region in 0–90° longitudinal band (similar to Fig. 1) distinctly stands out in the map. Therefore data before July 21, 2016 can be useful in some qualitative assessment in future. Due to malfunction of the instrument, not enough IR1 data is available to track either regions with high temperature or seasonal variation of surface temperatures.

## Conclusions

Venus surface temperatures indicate a hot (~698 K) with little to almost no interaction with solar energy. Assuming a solar constant of 2600 Wm^−2^, and 2.5% absorption by the surface the dayside temperature would be higher by about 1–2 K than nightside temperature. This indicates that the dayside surface temperatures would not be significantly different than that of the nightside surface temperatures. Surface temperature also show some dependency on altitude, and higher altitude regions are relatively colder than low altitude regions. However, due to various other physical processes such as hot-spot volcanism, lithospheric subduction, heat conduction, and solar insolation the surface temperatures do not show any strong correlation with altitude. Also due to major resurfacing, most of the Venus surface (70–80%) consists of flat plains indicating low dependency on atmospheric adiabatic lapse rate. Due to lack of heat conductivity/capacity data of Venus surface, the exact reasons/mechanisms for observed temperature distributions could not be determined.

Due to contamination from dayside during initial days, and instrument malfunction later it is not possible to study surface temperature variability in detail. With availability of more data from current mission (if the instrument comes back online in future) or from future missions, we can track some specific features and have better understanding of surface evolution.

## Methods

According to Planck’s law, spectral radiance of a blackbody at absolute temperature T for a given wavelength λ is given as:

2 $$L(\lambda ,T)=\frac{{c}_{1}}{{\lambda }^{5}[exp({c}_{2}/\lambda T)-1]}$$

where, *c*_1_ is equal to 2*hc*^2^, *h* (= 6.6260755 × 10^−34^ Js) is Planck’s constant, ‘*c*’ is the speed of light; *c*_2_ is equal to *hc/k*, *k* (=1.380658 × 10^−23^ J/K) is Boltzmann constant^24^. According to Eq. (2), the absolute temperature of a blackbody can be given as:

3 $$T(\lambda ,L)=\frac{{c}_{2}}{\lambda [\mathrm{ln}({c}_{1}/{\lambda }^{5}L)+1]}$$

1.01 μm being an almost perfect atmospheric window^7^, the IR1 measurements are almost unaffected by Venus atmospheric gases or clouds. This leads to a non-significant error in surface temperature estimation using radiance measurements from IR1 with Eq. (3).

Akaktsuki was successfully inserted into Venusian orbit on December 07, 2015, and start obtaining data since then^25^. However, due to malfunction of the electronics, the data acquisition by IR1 have stopped since December 07, 2016^6^. Initially, the nightside images acquired signal contamination from dayside due to charge overflowed from the dayside. Since July 21, 2016 the acquisition was corrected with specific planning. However, this lead to another kind of contamination due to instrumental bias^6^.

I have collected usable IR1 images from 2016, and divided them into two subsets, one prior to July 21, 2016 and second post July 21, 2016. I use radiance measurements from IR1 to retrieve absolute surface temperatures using Eq. (2). Finally, the retrieved temperatures are gridded into equal bins of 0.5° × 0.5° globally. For nightside, IR1 camera has a noise level of 1.3 μW cm^−2^ str^−1^ μm^−1^ at 260 K^5^, which leads to an error of less than 0.5 K in retrievals. A 5% change in observed radiance due to atmosphere would cause an error of about 2 K in surface temperature retrievals.

## Data Availability

Akatsuki IR1 data available at https://darts.jaxa.jp/planet/project/akatsuki/ir1.html.en. Magellan altimeter data available at http://pds-geosciences.wustl.edu/missions/magellan/gxdr/. High resolution gridded topography map from Magellan data is available at https://astrogeology.usgs.gov/search/map/Venus/Magellan/RadarProperties/Venus_Magellan_Topography_Global_4641m_v02. Data used to generate maps in this paper can be obtained by directly contacting the corresponding author.
